# Pharmacist involvement in antifungal stewardship programs: a systematic review of clinical, utilization, and economic outcomes

**DOI:** 10.1007/s11096-026-02149-5

**Published:** 2026-04-24

**Authors:** Emre Kara, Nese Sahin, Fatma Gul Yumrucu, Gokhan Metan

**Affiliations:** 1 Department of Clinical Pharmacy, Hacettepe University Faculty of Pharmacy, Altindag, Ankara, Turkey; 2 Hacettepe University Faculty of Pharmacy, Ankara, Turkey; 3 Department of Infectious Diseases and Clinical Microbiology, Hacettepe University Faculty of Medicine, Ankara, Turkey

**Keywords:** Antifungal agents, Antifungal stewardship, Antimicrobial stewardship, Inpatients, Mycoses, Pharmacists

## Abstract

**Introduction:**

Antifungal stewardship programs are increasingly implemented to optimize antifungal use. Pharmacists are frequently involved in these programs; however, their specific contributions and impact have not been systematically characterized.

**Aim:**

The aim of this systematic review was to synthesize the available evidence regarding the role and impact of pharmacists in antifungal stewardship programs, specifically focusing on clinical, utilization, and economic outcomes, using a narrative synthesis approach.

**Method:**

A systematic review was conducted. PubMed/MEDLINE and Scopus databases were searched for studies published from database inception to January 2026. Two reviewers independently screened titles, abstracts, and full texts according to predefined eligibility criteria. Data extraction was performed independently using a standardized form. Due to heterogeneity in study designs and outcomes, a narrative synthesis approach was applied. The review was reported in accordance with the PRISMA reporting guidelines. Risk of bias was assessed using the ROBINS-I tool for non-randomized studies.

**Results:**

Fifteen studies met the inclusion criteria, the majority of which were quasi-experimental pre–post intervention studies conducted in tertiary care hospitals. Key pharmacist-driven interventions included antifungal dose optimization, de-escalation or discontinuation of therapy, facilitation of early appropriate antifungal treatment, therapeutic drug monitoring, and education or protocol development. Across studies, pharmacist involvement was associated with improvements in antifungal prescribing quality and stewardship process outcomes, including potential improvements in duration of antifungal therapy, antifungal consumption (measured by defined-daily-doses or days-of-therapy), time to appropriate therapy, and antifungal-related costs. Effects on clinical outcomes such as mortality and length of hospital stay were variable and generally not statistically significant. The overall certainty of the evidence is low to very low by the observational nature and the moderate-to-serious risk of bias of the included studies.

**Conclusion:**

Pharmacist participation in multidisciplinary antifungal stewardship teams may be associated with improved antifungal utilization and adherence to guidelines in hospitalized adults. The findings support that pharmacists are important members of multidisciplinary teams in antifungal stewardship.

**Supplementary Information:**

The online version contains supplementary material available at 10.1007/s11096-026-02149-5.

## Impact statements

- This systematic review summarizes the available evidence on pharmacist involvement in antifungal stewardship programs and indicates potential improvements in antifungal utilization and stewardship-related process outcomes.
- Pharmacists frequently contribute to activities such as dose optimization, therapeutic drug monitoring (TDM), and integration of rapid diagnostic tests into clinical decision-making.
- The findings support the inclusion of clinical pharmacists as members of multidisciplinary antifungal stewardship teams to improve prescribing practices and antifungal management.
- Further well-designed studies are needed to better define the independent impact of pharmacist-led antifungal stewardship interventions.

## Introduction

Invasive fungal infections (IFIs) are defined as those involving sterile body sites such as the bloodstream, central nervous system, or organs such as the lungs, liver, and kidneys. Risk factors for IFIs include immunosuppression, hematologic malignancies, hematopoietic stem cell transplantation (HSCT), solid organ transplantation (SOT), critical illness, steroid treatment, diabetes mellitus, Coronavirus disease 2019 (COVID-19), influenza, and invasive devices such as central venous catheters (CVCs) [1, 2]. Over the past 20 years, the incidence of IFIs has increased significantly [3]. Despite the increasing prevalence of diseases caused by rare fungal species worldwide, the majority of invasive fungal infections are still caused by *Candida, Aspergillus,* and *Cryptococcus* species [4].

Treatment options for IFIs are limited. The widely available systemic antifungal agents include polyenes, azoles, echinocandins, and flucytosine [1, 5]. The development of interventions by multidisciplinary teams is recommended to optimize the use of available therapeutic agents [6, 7]. Antifungal therapy is challenging due to antifungal resistance, drug-drug interactions, and adverse effects [8, 9].

The selection of appropriate antifungal medication and the determination of the treatment duration are both complex processes that are influenced by several factors [10]. Prolonged antifungal treatment results in a longer hospital stay and higher treatment costs [11]. To combat drug resistance and improve patient outcomes, appropriate antifungal use is essential. Implementing antifungal stewardship programs is therefore crucial to monitor and promote optimal prescribing practices [12].

Pharmacists are the key members of antimicrobial stewardship teams. The role of pharmacists in antifungal stewardship programs has increasingly been investigated in recent years. Although antifungal stewardship programs are increasingly implemented, the specific contributions of pharmacists within these programs have not been comprehensively synthesized.

### Aim

The aim of this systematic review was to synthesize the available evidence regarding the role and impact of pharmacists in antifungal stewardship programs, specifically focusing on clinical, utilization, and economic outcomes, using a narrative synthesis approach.

## Method

This systematic review was reported in accordance with the Preferred Reporting Items for Systematic Reviews and Meta-Analyses (PRISMA) guidelines (https://www.prisma-statement.org/). The review protocol, including the research strategy, was registered in the International Prospective Register of Systematic Reviews (PROSPERO) (CRD488701). No major deviations from the registered protocol occurred.

### Search strategy

A systematic literature search was conducted in accordance with the Preferred Reporting Items for Systematic Reviews and Meta-Analyses (PRISMA) guidelines. PubMed/MEDLINE and Scopus databases were systematically searched from database inception to January 1, 2026. Although EMBASE and CINAHL databases were not accessible at our institution at the time of the search, the selected databases provide broad coverage of biomedical literature. To minimize the risk of missing relevant studies, the reference lists of all included studies and relevant review articles were manually screened. However, grey literature sources and trial registries were not searched in this review. The detailed search strategies for each database are provided in the Supplementary Material.

The search strategy employed a combination of keywords related to the intervention (“stewardship,” “pharmacist”) and condition (“antifungal agents,” “mycoses,” “fungal infection”). Eligibility was restricted to randomized, quasi-experimental, before–and–after, retrospective, and longitudinal observational studies evaluating antifungal stewardship interventions in adult inpatient populations. Abstracts from conferences, non-interventional studies, patients receiving topical antifungal treatment, and studies conducted on outpatients and pediatric patients were excluded. The analysis was limited to articles in English.

### Study selection and data extraction

Two researchers conducted a literature search and screened the titles and abstracts of identified studies for eligibility according to the inclusion criteria. The online systematic review screening and data extraction tool, the Covidence website (https://app.covidence.org/), was used to store, categorize, and resolve any conflicts between investigators regarding the inclusion or exclusion of studies. When there was insufficient information to assess the eligibility of abstracts, the full text of the articles was reviewed. Two reviewers independently conducted the study selection and data extraction processes, with disagreements resolved through discussion or consultation with a third reviewer. Subsequently, full-text articles were reassessed to confirm their eligibility according to the predefined inclusion criteria. The two researchers then met again to review the controversial articles. They selected appropriate articles for data extraction. In case of disagreement at this stage, the third researcher ensured that the final decision was made.

The research question and eligibility criteria were structured according to the PICO framework. The population (P) included hospitalized adult patients receiving antifungal therapy or managed within antifungal stewardship programs. The intervention (I) consisted of pharmacist-led or pharmacist-involved antifungal stewardship interventions. The comparator (C) included usual care, periods before implementation of stewardship interventions, or settings without pharmacist involvement. The outcomes (O) included antifungal utilization metrics (e.g., duration of therapy, antifungal consumption), stewardship process indicators, clinical outcomes and economic outcomes (e.g., antifungal acquisition costs, stewardship-related savings) where reported.

The data were extracted on the following variables: study methods, setting, patient population, method of patient identification, antifungal stewardship team members, interventions, pharmacist role, performance, and outcome measures (antifungal drug use, antifungal prescription quality, and clinical outcomes). Clinical outcomes of interest included all-cause mortality, length of hospital stay, and intensive care unit (ICU) length of stay. These outcomes were defined a priori by the review authors but were only extracted when reported in the included studies.

### Risk of bias assessment

Risk of bias for all included studies was assessed using the Risk of Bias in Non-randomized Studies of Interventions (ROBINS-I) tool. After study selection, it became evident that most included studies were non-randomized and quasi-experimental in design. Therefore, the ROBINS-I tool was selected as the most appropriate instrument for assessing risk of bias in non-randomized studies of interventions. Seven domains were evaluated: bias due to confounding, selection of participants, classification of interventions, deviations from intended interventions, missing data, measurement of outcomes, and selection of the reported result.

Two reviewers independently assessed the risk of bias for each included study. Discrepancies were resolved through discussion and consensus. Overall risk of bias for each study was determined according to the highest level of risk attributed to any individual domain, in accordance with ROBINS-I guidance. Risk of bias assessments were summarized both in tabular format and visually using the robvis visualization tool [13].

### Data synthesis

Due to heterogeneity in study designs, interventions, and reported outcomes, a meta-analysis was not considered appropriate. Therefore, a narrative synthesis approach was used to summarize the findings of the included studies. The results were synthesized by grouping studies according to the type of pharmacist involvement in antifungal stewardship interventions and the outcomes reported, including antifungal utilization metrics, stewardship process indicators, and clinical outcomes where available.

## Results

### Study characteristics

**Fig. 1:**
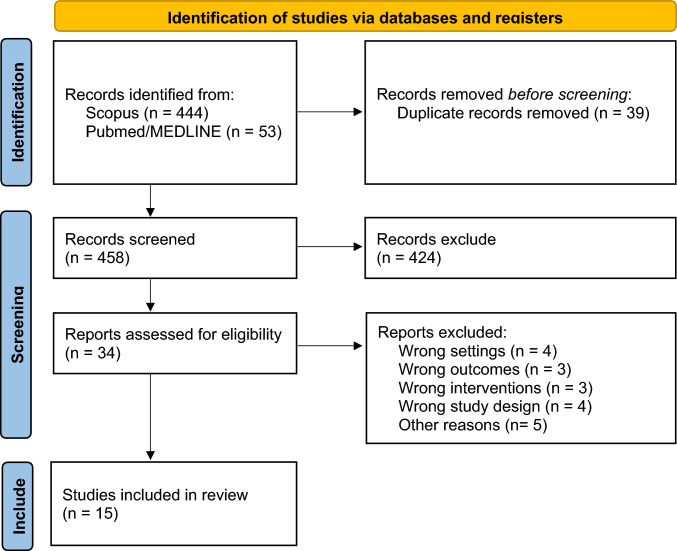
Identification, screening, and inclusion stages of the reviewed articles and flow chart of selected studies

A total of 497 studies were identified during the initial screening. Following the removal of duplicates, 458 articles remained for title and abstract screening. Of these, 424 articles were excluded as they were not relevant to the research question. The full text of 34 articles was reviewed. Following a comprehensive review of all articles, 19 articles were excluded for reasons detailed in the PRISMA flow diagram (Fig. 1). Fifteen studies published between 2012 and 2023 met the inclusion criteria for this systematic review (Fig. 1). All included studies evaluated antifungal stewardship interventions implemented in adult hospitalized patients, predominantly within tertiary care or academic medical centers. The most common methodological approach was quasi-experimental design (n = 7), including pre–post and before–and-after interventions. Additional study designs included longitudinal analyses (n = 2), observational interventions (n = 2), retrospective studies (n = 1), interrupted time-series analyses (n = 1), and other interventional designs (Table 1).

**Table 1:** Characteristics of studies included in the systematic review

Study	Country	Study design	Setting	Patient population	Sample size	Study duration	Antifungal agents evaluated	Comparator	Primary stewardship focus
Standiford et al. [26]	USA	Longitudinal analysis	Academic medical center	All adult patients receiving antifungal therapy	Not reported	10 years (2001–2010)	All systemic antifungals	Periods before and after implementation of stewardship activities across the study program	Cost-focused ASP
Cappelletty and Jacobs [28]	USA	Before–after comparison	Academic medical center	Adult inpatients receiving micafungin, linezolid, or imipenem-cilastatin for at least 72 h	230 patients	15 months	Micafungin	Periods with pharmacist involvement versus periods without pharmacist involvement	Pharmacist absence analysis
Reed et al. [15]	USA	Pre–post intervention	Tertiary care hospital	All adult patients receiving antifungal therapy	173 patients	24 months	All systemic antifungals	Usual care before implementation of candidemia-focused stewardship interventions	Candidemia management
Al-Somai et al. [21]	Saudi Arabia	Retrospective study	Tertiary care hospital	Adult inpatients receiving caspofungin, imipenem, or meropenem	357 patients	1 year	Caspofungin	Usual care before clinical pharmacist and infectious diseases intervention	Pharmacist-ID consultant collaboration
Whitney et al. [23]	UK	Longitudinal intervention	Tertiary care hospital	All adult patients receiving antifungal therapy	432 patients	6 years	All systemic antifungals	Pre-intervention period before implementation of the antifungal stewardship program	Hospital-wide AFS program
Pettit et al. [16]	USA	Quasi-experimental	Tertiary care hospital	Adult inpatients with candidemia	84 patients	2 years	All systemic antifungals	Usual care before automated candidemia alerts and stewardship review	Automated candidemia alerts
Morris et al. [27]	Canada	Interrupted time series	Academic ICUs	All adult patients receiving antifungal therapy	57,195 patients	9 years	All systemic antifungals	Pre-implementation period before phased antimicrobial stewardship intervention	Long-term ASP impact
Lachenmayr et al. [20]	Germany	Quasi-experimental (pre–post)	Tertiary care hospital	Adult inpatients hospital with a diagnosis of hematological or oncological malignancy	207 patients	1 year	All systemic antifungals	Pre-intervention period before implementation of antifungal stewardship interventions	Optimization of antifungal prescribing
Samura et al. [19]	Japan	Quasi-experimental	Acute care hospital	Adult inpatients with candidemia	37 patients	8 years	All systemic antifungals	Pre-intervention period before pharmacist-led antifungal stewardship activities	Pharmacist-led AFS activities
Koh et al. [14]	USA	Pre–post intervention	Academic medical center	Adult inpatients with candidemia	264 patients	15 months	All systemic antifungals	Pre-intervention period before incorporation of rapid diagnostic testing and stewardship intervention	Rapid diagnostics–guided therapy
Ioannidis et al. [25]	Greece	Observational intervention	Hospital setting	All adult patients receiving antifungal therapy	157 patients	6 years	All systemic antifungals	Baseline period before implementation of the antifungal stewardship program	Antifungal stewardship adoption
Kara et al. [18]	Turkiye	Quasi-experimental (pre–post)	Tertiary care hospital	All adult patients receiving antifungal therapy	377 patients	21 months	All systemic antifungals	Pre-intervention period before pharmacist-driven antifungal stewardship implementation	Pharmacist-driven antifungal stewardship
Markogiannakis et al. [24]	Greece	Pre–post intervention	Tertiary care hospital	All adult patients receiving antifungal therapy	285 patients	20 months	All systemic antifungals	Pre-intervention period before implementation of the antifungal stewardship program	Lower antifungal overuse
Moni et al. [17]	India	Quasi-experimental	Tertiary care hospital	Adult inpatients with candidemia	175 patients	6 years	All systemic antifungals	Pre-bundle period before implementation of the candidemia care bundle	Candidemia care bundle
Keck et al. [22]	USA	Quasi-experimental	Academic medical center	Adult inpatients receiving at least one dose of micafungin	282 patients	18 months	Micafungin	Pre-intervention period before the micafungin-focused antifungal stewardship initiative	Targeted echinocandin stewardship

ASP, antimicrobial stewardship program; AFS, antifungal stewardship; ID, infectious disease; ICU, intensive care unit

This systematic review included 15 studies investigating the role of the pharmacist in antifungal stewardship. The studies were published between 2012 and 2023, with no eligible studies identified for the years 2024–2026 at the time of the search and a clear increase in publications from 2019 onward.

The primary focus of the stewardship interventions varied, reflecting an evolution in antifungal stewardship strategy. While earlier studies often emphasized cost analysis and program adoption, more recent investigations centered on specific, pharmacist-driven activities, such as managing candidemia through care bundles or rapid diagnostics, optimizing prescribing in high-risk populations, and implementing targeted stewardship for drug classes like echinocandins. (Table 1).

The studies included various healthcare professionals as members of the antifungal stewardship team, including clinical pharmacists, hospital pharmacists, infectious disease physicians, microbiologists, radiologists, and nurses. However, in seven out of 15 studies, the antifungal stewardship team consisted of an infectious disease physician and a pharmacist (Table 2).

**Table 2:** Characteristics of antifungal stewardship interventions

Study	Stewardship team	Key pharmacist-led activities	Intervention type	Specific pharmacist activities
Standiford et al. [26]	ID physician + clinical pharmacist	Cost-focused stewardship	Program-level AFS	Reviewing antifungal orders for policy compliance, eliminating redundant coverage, facilitating IV-to-oral conversion where clinically appropriate, and referring complex cases to infectious diseases specialists
Cappelletty and Jacobs [28]	ID physician + clinical pharmacist	Evaluation of pharmacist absence	System impact	Prospective identification of patients on restricted antimicrobials, with pharmacist-initiated review within 72–96 h guided by culture findings and institutional protocols, in collaboration with the ID physician as warranted
Reed et al. [15]	ID physician + pharmacist + microbiologist	Early appropriate therapy optimization	Diagnostic-driven	Contacting the treating physician to initiate antifungal therapy when not yet prescribed, and coordinating ID and ophthalmology referrals and central venous catheter removal per candidemia management recommendations
Al-Somai et al. [21]	Pharmacist + ID physician	Rationalization of antifungal use	Pharmacist-led AFS	CP reviewed medical chart, lab tests and culture reports and provided therapeutic interventions as needed to the treating physicians regarding doses, interactions and duration of antimicrobial therapy
Whitney et al. [23]	ID consultant + antimicrobial pharmacist	Institutional antifungal policies	Program-level AFS	All patients identified were seen on a weekly stewardship ward round by an infectious diseases consultant and antimicrobial pharmacist, which incorporated reviewing medical notes, drug charts, laboratory tests and imaging
Pettit et al. [16]	Pharmacist + IT + ID physician	Automated candidemia alerts	Diagnostic-driven	ASP pharmacist reviews automated EMR (Epic) alerts for candidemia patients daily; conducts chart review; contacts primary team to recommend appropriate antifungal initiation, ID consultation, CVC removal, repeat blood cultures, and ophthalmology consultation per bundle-of-care criteria
Morris et al. [27]	ID physician + pharmacist	Long-term ASP implementation	System impact	NS
Lachenmayr et al. [20]	ID specialists + clinical pharmacist	Guideline implementation, therapy optimization	Program-level AFS	Every antifungal prescription was evaluated by a clinical pharmacist on a daily basis (Mon–Fri) followed by feedback to the prescribing physicians
Samura et al. [19]	Pharmacist + physicians	Daily antifungal review, de-escalation	Pharmacist-led AFS	An ID pharmacist embedded in the pharmacy department received blood culture reports and intervened promptly in bacteremia/fungemia cases. Ward pharmacists provided dosing and treatment recommendations from the outset, working in tandem with the ID pharmacist. A structured consultation pathway was established for severe infections, with the ID pharmacist also liaising with a part-time ID physician for refractory cases
Koh et al. [14]	Pharmacist + microbiology + ID physician	Rapid diagnostic–guided therapy	Diagnostic-driven	Unit-based clinical pharmacists conducted daily appropriateness reviews of antifungal/antibiotic therapy for all patients with positive BioFire panel results and provided prescriber recommendations as needed
Ioannidis et al. [25]	ASP team (including an infection control specialist and a clinical pharmacist)	Non-compulsory AFS interventions	Program-level AFS	NS
Kara et al. [18]	ID specialists + clinical pharmacists + clinical microbiologists + radiologist	Candidemia care bundle implementation	Bundle-based AFS	Clinical pharmacists (CPs) conducted daily ward rounds across ICU, internal medicine, oncology, and HSCT units, evaluating antifungal regimens for indication, agent selection, dosing, route, and potential drug–drug interactions in accordance with institutionally adapted international guidelines
Markogiannakis et al. [24]	Pharmacist + ID physician	Prospective audit, dose optimization, TDM	Pharmacist-led AFS	Cessation of unjustified prophylaxis or empiric therapy, stepdown of antifungal treatment when clinically feasible, and integration of fungal biomarkers and TDM into prescribing decisions
Moni et al. [17]	Physician/hospitalist + intensivist + microbiologists + clinical pharmacists + administrative champion	Review of antifungal indications	Program-level AFS	Systematic reassessment of antifungal prescriptions against a five-domain framework encompassing indication, agent, dose, frequency, and treatment duration
Keck et al. [22]	Antimicrobial stewardships + ID trained pharmacists + ID pharmacy resident	Targeted micafungin restriction	Pharmacist-led AFS	Prospective audit and feedback interventions targeting antifungal de-escalation, therapy discontinuation, and infectious diseases consultation

ASP, antimicrobial stewardship program; AFS, antifungal stewardship; ID, infectious disease; IT, information technology; TDM, therapeutic drug monitoring; NS, not specified

### Risk of bias assessment

The risk of bias assessment for the included studies is summarized in Supplementary Table S1 and visualized in Supplementary Figure S1. Overall, most studies were judged to have a moderate risk of bias, primarily due to their observational and pre–post intervention designs. The most common concerns were related to confounding and the selection of participants. Bias related to outcome measurement and missing data was generally considered low across studies.

### Characteristics of antifungal stewardship interventions

The antifungal stewardship interventions evaluated across the included studies were heterogeneous in design and scope but shared common structural elements. Interventions were generally multidisciplinary, most frequently involving collaboration between pharmacists, infectious disease physicians, clinical microbiologists, and infection control teams. However, pharmacists frequently contributed to the day-to-day implementation of stewardship activities.

Interventions could be broadly categorized into diagnostic-driven strategies, pharmacist-led prospective audit and feedback, and program-level antifungal stewardship initiatives. Diagnostic-driven interventions were implemented in four studies, incorporating rapid diagnostic tests, automated electronic alerts, or microbiology-triggered notifications to facilitate earlier identification of candidemia and initiation of appropriate antifungal therapy [14–17]. Pharmacist-led interventions were reported in five studies and most commonly included daily antifungal therapy review, dose optimization based on renal function or pharmacokinetic principles, therapeutic drug monitoring, de-escalation or discontinuation of unnecessary therapy, and structured education of prescribers [18–22]. Program-level antifungal stewardship initiatives were described in four studies and focused on the implementation of institutional stewardship policies, standardized treatment algorithms, and bundled care approaches at the hospital level [23–26].

### Impact on antifungal utilization

Lower antifungal utilization was reported in 9 of the 15 included studies following implementation of antifungal stewardship interventions. Decreases in overall antifungal consumption were most commonly measured using defined daily doses or days of therapy (DOT) [18–26].

Reductions were most frequently observed for echinocandins and for prolonged empirical antifungal therapy, particularly in studies implementing targeted stewardship interventions or pharmacist-led review strategies.

In addition to overall consumption, improvements in the appropriateness of antifungal prescribing were reported in eight studies. These included reductions in unnecessary antifungal initiation, earlier discontinuation of therapy in patients without confirmed fungal infection, and increased use of targeted therapy based on microbiological data [14–20, 22] (Supplementary Table S2).

### Clinical outcomes

Clinical outcomes were reported in 12 of the 15 included studies and were generally not the primary endpoints of antifungal stewardship interventions. Among these studies, eleven reported no statistically significant differences in all-cause mortality between pre- and post-intervention periods [14–16, 18, 19, 21–25, 27] and one reported an increase in mortality [20].

Length of hospital stay was evaluated in 11 studies, with nine demonstrating no significant change [15–17, 19, 20, 22, 24–26], one reporting a modest reduction [27], and one reporting an increase following stewardship implementation [18]. Intensive care unit duration was assessed in a limited number of studies and showed heterogeneous results, likely influenced by patient severity and comorbidities rather than antifungal management alone.

In contrast to patient-centered clinical outcomes, antifungal stewardship interventions impacted antifungal-related outcomes. Lower antifungal consumption or duration of therapy were observed in eight studies, and decreased use of broad-spectrum agents, particularly echinocandins, was reported in several pharmacist-led and program-level interventions [18–24, 26].

Cost-related outcomes were evaluated in seven studies, with six demonstrating reductions or no significant changes following intervention implementation [18, 19, 22–24, 26] and one reporting an increase in antifungal-related costs [15] (Supplementary Table S2).

### Stewardship-related process outcomes

Stewardship-related process outcomes demonstrated the most consistent and robust improvements across the included studies. Interventions were associated with lower time to appropriate antifungal therapy, increased adherence to guideline-recommended management practices, and improved compliance with candidemia care bundles.

Several studies also reported improvements in documentation quality, antifungal indication review, and follow-up blood culture monitoring. These findings highlight the effectiveness of antifungal stewardship interventions in enhancing the quality and timeliness of antifungal decision-making, even in the absence of consistent effects on mortality or length of hospital stay.

Twelve studies reported their outcomes related to the duration of treatment [14–22, 24, 25, 28]. Six studies indicate a lower duration of treatment [14–17, 19, 22]. Seven studies provided information regarding the dosing of antifungal medications [19–21, 23–25, 27] (Supplementary Table S2).

### Role of pharmacists in antifungal stewardship programs

The most robust finding across the published studies is the pharmacist’s central role in reducing inappropriate antifungal use. Through prospective audit and feedback [17, 24], implementation of clinical guidelines [20], and targeted drug stewardship initiatives [22], pharmacists directly decrease overuse, misuse, and the overall duration of antifungal therapy.

Beyond volume reduction, pharmacists improve the precision and timeliness of antifungal therapy. They are pivotal in integrating rapid diagnostic tests to shorten the time to appropriate therapy [14] and are instrumental in ensuring adherence to evidence-based candidemia care bundles [16, 18], thereby standardizing best practices and improving process outcomes (Table 3).

**Table 3:** Pharmacist-involved antifungal stewardship interventions and outcomes

Study	Key pharmacist intervention	Outcome category	Direction of effect
Standiford et al. [26]	Cost-focused ASP	Cost	↓ Antifungal expenditure (45.8% decrease) (*p* = 0.003)
Cappelletty and Jacobs [28]	Evaluation of pharmacist absence	System performance	↑ Stewardship effectiveness (54% vs. 84.6%) (*p* = N/A)
Reed et al. [15]	Early therapy optimization	Process	↑ Effective antifungal therapy (88% vs. 99%) (*p* = 0.008)
Al-Somai et al. [21]	Pharmacist–ID intervention	Utilization	↓ Inappropriate antifungal use (*p* = N/A)
Whitney et al. [23]	Program-level AFS	Utilization, cost	↓ Antifungal use (26% decrease), ↓ costs (30% decrease) (*p* = N/A)
Pettit et al. [16]	Automated alerts with pharmacist review	Process	↑ Bundle adherence (48% vs. 83%) (*p* = 0.001)
Morris et al. [27]	Long-term ASP implementation	System outcomes	↓ Antifungal use (− 3.16 defined daily dose/100 patient-days) (*p* = 0.005)
Lachenmayr et al. [20]	Guideline-based therapy optimization	Utilization	↑ Dosage accuracy (+ 19.3%), correct choice of drug (+ 15.9%), ↓ potential clinically relevant drug–drug interactions with concomitant medication (− 13.9%) (*p* < 0.05),
Samura et al. [19]	Pharmacist-led antifungal review	Utilization, cost	↓ DOT (6 vs. 3.4) (*p* < 0.001), ↓ antifungal cost (9390.5 ± 5687.1 vs. 5930.8 ± 4687.0 US dollars) (*p* = 0.002)
Koh et al. [14]	RDT-triggered pharmacist intervention	Process	↓ Time to optimal therapy [53.7 (57.7) vs. 38.4 (31.5) hours] (*p* < 0.001)
Ioannidis et al. [25]	Non-compulsory AFS program	Utilization	↓ Antifungal overuse (*p* = N/A)
Kara et al. [18]	Pharmacist-supported care bundle	Process	↑ Overall appropriateness of antifungal use (27.8%, 32.4%, and 71.9%) (*p* < 0.001)
Markogiannakis et al. [24]	Prospective audit, dosing optimization, TDM	Utilization, process	↓ Inappropriate use (36.9% vs. 16.6%) (*p* < 0.001)
Moni et al. [17]	Stewardship review of prescriptions	Utilization	↑ Appropriateness of antifungal prescriptions (30% vs. 65%) (*p* < 0.001)
Keck et al. [22]	Targeted echinocandin stewardship	Utilization	↓ Echinocandin exposure (4 [IQR 3–6] in vs. 3 [IQR 2–6]) (*p* = 0.005)

ASP, antimicrobial stewardship program; AFS, antifungal stewardship; ID, Infectious disease; DOT, days of therapy; TDM, therapeutic drug monitoring; RDT, rapid diagnostic tests

## Discussion

In this systematic review of 15 interventional studies, we evaluated the impact of pharmacist-led and pharmacist-involved antifungal stewardship interventions in adult hospitalized patients. The findings suggest that pharmacist participation in antifungal stewardship is frequently associated with improvements in antifungal utilization and stewardship-related process outcomes, whereas effects on patient-centered clinical outcomes such as mortality remain variable. These observations were reported across tertiary care and academic hospital settings where most antifungal stewardship programs are implemented [18–20, 23]. It is important to note that the interventions described were largely collaborative. Therefore, the observed improvements should be interpreted as successes of the multidisciplinary stewardship team, in which the pharmacist plays a pivotal role, rather than purely independent pharmacist effects.

The findings of this systematic review demonstrate substantial heterogeneity among the included studies. This variability primarily is derived from clinical and methodological differences across the literature. The studies ranged from pre-post observational designs to interrupted time-series analyses, with varying durations of follow-up. The patient populations were highly diverse, spanning from high-risk hematology-oncology units to general medical wards, each with different antifungal utilization patterns. Furthermore, the scope of pharmacist-led interventions showed considerable diversity, encompassing activities from bedside clinical consultations and dose optimization to administrative stewardship audits and guideline development. These discrepancies precluded a formal meta-analysis and justified the use of a narrative synthesis approach.

The importance of optimizing antifungal use is underscored by the rising burden and complexity of invasive fungal infections, which are associated with substantial morbidity, mortality, and healthcare utilization in hospitalized adults [29]. Inappropriate empirical antifungal therapy, delays in targeted treatment, and prolonged exposure to broad-spectrum agents remain common challenges in routine practice. Within this context, antifungal stewardship represents a critical strategy to balance timely treatment with avoidance of unnecessary antifungal exposure.

A key finding of this review is the consistency of improvements in antifungal utilization, particularly lower unnecessary or prolonged antifungal therapy and decreased use of broad-spectrum agents such as echinocandins. Multiple studies reported lower antifungal consumption following implementation of pharmacist-driven or multidisciplinary antifungal stewardship interventions, including targeted echinocandin stewardship and guideline-based prescribing optimization [18, 20, 22–24]. Our findings are broadly consistent with a previous systematic review of antimicrobial stewardship programs, which have also reported reductions in antimicrobial use and healthcare costs but limited evidence for improvements in mortality outcomes. The present review differs from the systematic review by Hart et al. in several important respects. Hart et al. included 13 US-based studies evaluating antifungal stewardship more broadly, without specifically requiring pharmacist involvement and without excluding pediatric populations. By contrast, our review focused specifically on adult hospitalized patients and on antifungal stewardship interventions in which pharmacists had a defined role. As a result, our review offers a more targeted perspective on pharmacist contribution to antifungal stewardship implementation, while Hart et al. provides a broader overview of antifungal stewardship interventions in general [30].

Some studies suggested that reduced pharmacist involvement may be associated with less favorable stewardship performance [28], although the independent effect of pharmacists could not be fully separated from the broader multidisciplinary intervention structure [27]. The study by Cappelletty & Jacobs evaluated outcomes in the absence of pharmacist involvement, which provides indirect evidence regarding the potential impact of pharmacist participation in stewardship programs [28]. Stewardship-related process outcomes emerged as the most robust and reproducible benefits of antifungal stewardship interventions. Improvements in time to appropriate antifungal therapy, adherence to candidemia management bundles, and acceptance of stewardship recommendations were observed, particularly in studies integrating pharmacist-led review with diagnostic triggers or automated alerts [14–17]. These findings are clinically meaningful, as timely initiation of appropriate antifungal therapy is strongly emphasized in consensus treatment guidelines for invasive yeast infections, particularly in high-risk populations such as critically ill, hematology, and oncology patients [31].

In contrast, effects on clinical outcomes such as mortality and length of hospital stay were inconsistent and generally not statistically significant. Several studies reported no difference in mortality despite improvements in antifungal prescribing quality [15, 16, 27], while others demonstrated improvements in intermediate outcomes without corresponding survival benefits [17]. This variability likely reflects the multifactorial determinants of outcomes in invasive fungal infections, including host comorbidities, severity of illness, source control, and diagnostic delays, which may not be directly modifiable through stewardship interventions alone. Importantly, most included studies were not powered to detect differences in mortality, and clinical outcomes were typically secondary endpoints.

Some studies reported reductions in antifungal drug expenditure following pharmacist-involved stewardship interventions [19, 26], suggesting a potential economic benefit of integrating pharmacists into antifungal stewardship programs. Pharmacists appeared to contribute substantially to the day-to-day implementation of antifungal stewardship activities across the included studies. Pharmacist responsibilities extended beyond medication review to include dose optimization, therapeutic drug monitoring, interpretation of microbiological data, coordination of diagnostic information, and real-time communication with prescribers [18, 19, 21]. The relevance of these activities is particularly evident in vulnerable patient populations, such as older adults, where complex pharmacokinetics, polypharmacy, and increased susceptibility to adverse drug events amplify the risks associated with inappropriate antifungal use [32]. Studies evaluating reduced pharmacist involvement or pharmacist absence demonstrated deterioration in stewardship performance, suggesting that pharmacist involvement may support the continuity and implementation of antifungal stewardship activities [27, 28].

Several studies incorporated diagnostic-driven stewardship strategies, including rapid diagnostic tests and automated candidemia alerts, which were most effective when coupled with pharmacist-led intervention [14, 16]. These findings emphasize that diagnostic technologies alone are insufficient to improve antifungal management unless paired with timely clinical interpretation and action. Pharmacists may be well placed to help integrate diagnostic information into therapeutic decision-making, particularly when stewardship interventions are linked to rapid diagnostic or alert-based systems.

This review focused exclusively on adult hospitalized populations, reflecting the structure of most antifungal stewardship programs and the epidemiology of invasive fungal infections in adult care settings. Pediatric populations were excluded due to fundamental differences in epidemiology, antifungal pharmacokinetics, and stewardship considerations, which have been addressed separately in the literature [33]. This focus enhances the applicability of the findings to adult stewardship practice but limits generalizability to pediatric settings.

Our study has several limitations. First, the majority of included studies were non-randomized and quasi-experimental, introducing potential bias and limiting causal inference. Second, heterogeneity in intervention design, outcome definitions, and reporting practices precluded quantitative synthesis. Third, the individual contribution of pharmacists could not always be isolated from broader multidisciplinary stewardship efforts. Fourth, the interpretation of our findings is constrained by the quality of the included literature. According to the ROBINS-I assessment, the majority of studies (11 of the 15) were at moderate to serious risk of bias, primarily due to their non-randomized, observational designs. Such risks suggest a high likelihood of overestimating the intervention effects, as confounding factors cannot be fully controlled. Fifth, a formal GRADE assessment was not conducted and a quantitative meta-analysis was not performed due to the heterogeneity of study designs and reported outcomes. Sixth, the exclusion of additional databases such as EMBASE and CINAHL may have limited retrieval of localized or smaller-scale studies that are less likely to be indexed in PubMed/MEDLINE or Scopus. Finally, formal inter-rater agreement statistics were not calculated. One of the included studies [18] includes the corresponding author of this manuscript. This study was assessed using the same eligibility criteria and risk-of-bias assessment as all other included studies. To ensure transparency, this has now been explicitly disclosed in the manuscript.

Despite these limitations, the findings of this review have important implications for clinical practice and policy. The overall pattern of findings suggests that pharmacist involvement may be beneficial within multidisciplinary antifungal stewardship programs, particularly in institutions with high antifungal use, complex patient populations, or limited infectious disease support.

## Conclusion

This systematic review suggests that pharmacist-involved antifungal stewardship interventions may improve antifungal utilization and stewardship-related process outcomes in adult hospitalized patients. Across the included studies, pharmacist participation was associated with more appropriate antifungal prescribing, reduced unnecessary antifungal exposure, and better adherence to recommended management practices.

However, effects on patient-centered clinical outcomes such as mortality and length of hospital stay remained variable and inconclusive. Overall, the available evidence supports pharmacist involvement as an important component of multidisciplinary antifungal stewardship programs, while highlighting the need for higher-quality studies to better define the independent contribution of pharmacists.

## Supplementary Information

Below is the link to the electronic supplementary material.

Supplementary file1 (DOCX 660 kb)

## Data Availability

No datasets were generated or analysed during the current study.
