# Supplementary material for: Pharmacist involvement in antifungal stewardship programs: a systematic review of clinical, utilization, and economic outcomes
Source: Int J Clin Pharm. 2026 Apr 24;48(4):1214–27. doi: 10.1007/s11096-026-02149-5 (PMC13369351; doi:10.1007/s11096-026-02149-5)
Supplement: Supplementary file 1 — Supplementary file1 (DOCX 660 kb) [file 11096_2026_2149_MOESM1_ESM.docx]

**Supplementary Table S1.** Risk of Bias Assessment of Included Studies (ROBINS-I)

| **Study** | **D1** | **D2** | **D3** | **D4** | **D5** | **D6** | **D7** | **Overall** |
| --- | --- | --- | --- | --- | --- | --- | --- | --- |
| **Standiford et al., 2012** | Serious | Low | Moderate | Moderate | Low | Low | Moderate | Serious |
| **Cappelletty & Jacobs, 2013** | Serious | Low | Moderate | Low | Low | Moderate | Moderate | Serious |
| **Reed et al., 2014** | Moderate | Low | Low | Moderate | Low | Low | Moderate | Moderate |
| **Al-Somai et al., 2014** | Serious | Moderate | Low | Moderate | Moderate | Moderate | Moderate | Serious |
| **Whitney et al., 2018** | Moderate | Moderate | Low | Moderate | Low | Low | Moderate | Moderate |
| **Pettit et al., 2019** | Moderate | Low | Low | Low | Low | Low | Low | Moderate |
| **Morris et al., 2019** | Serious | Low | Low | Moderate | Low | Low | Moderate | Serious |
| **Lachenmayr et al., 2019** | Moderate | Low | Low | Moderate | Low | Moderate | Moderate | Moderate |
| **Samura et al., 2020** | Moderate | Low | Low | Moderate | Low | Low | Moderate | Moderate |
| **Koh et al., 2020** | Moderate | Low | Low | Moderate | Low | Low | Moderate | Moderate |
| **Ioannidis et al., 2020** | Serious | Moderate | Low | Moderate | Moderate | Moderate | Moderate | Serious |
| **Kara et al., 2021** | Moderate | Low | Low | Low | Low | Low | Low | Moderate |
| **Markogiannakis et al., 2021** | Moderate | Low | Low | Moderate | Low | Low | Moderate | Moderate |
| **Moni et al., 2022** | Moderate | Low | Low | Moderate | Low | Low | Moderate | Moderate |
| **Keck et al., 2023** | Moderate | Moderate | Low | Moderate | Low | Low | Moderate | Moderate |

**
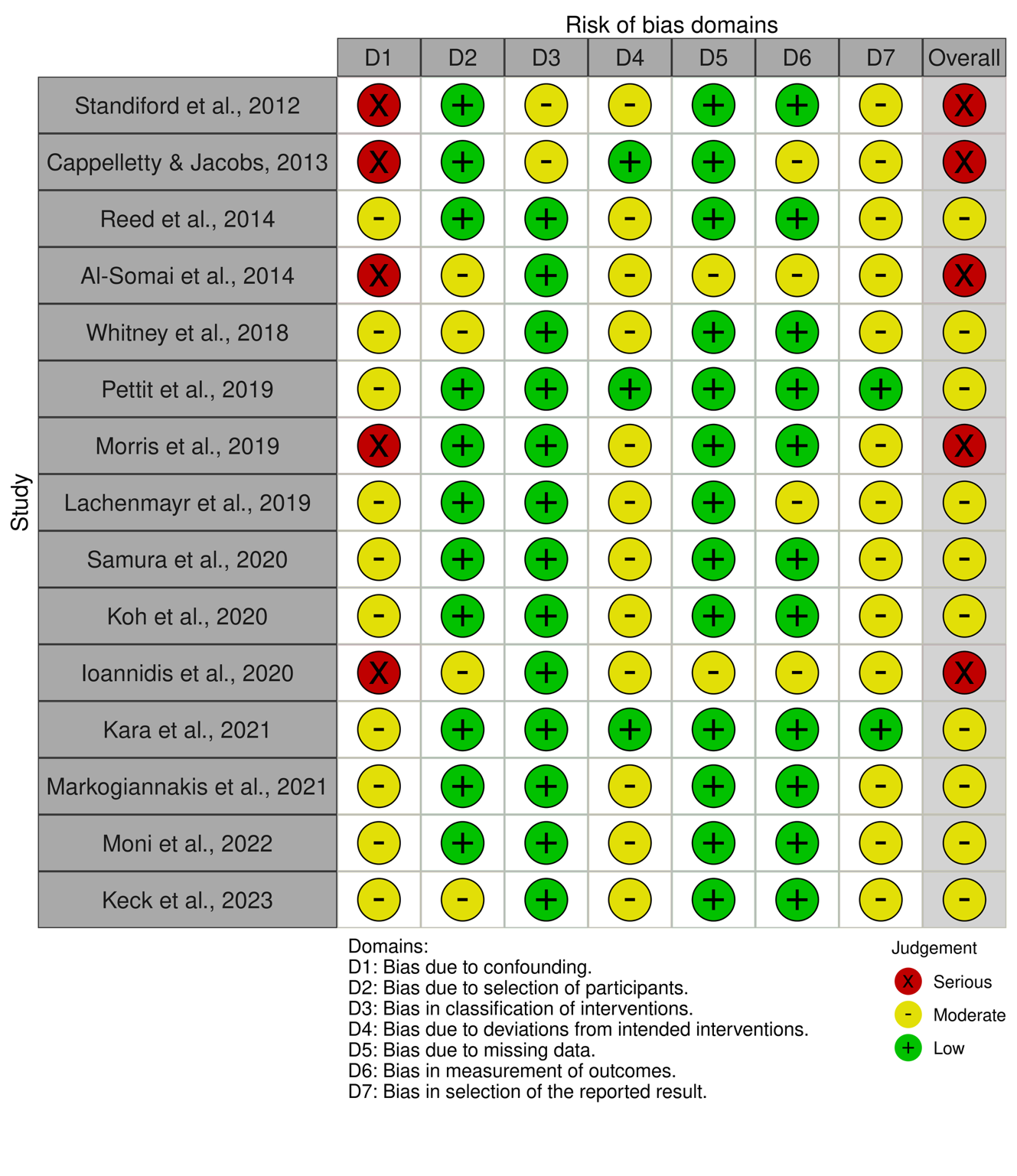
**

**Supplementary Figure S1.** Risk of Bias Assessment of Included Studies (ROBINS-I)

**Supplementary Table S2.** Results of Antifungal Stewardship Interventions

| **Reference** | **Mortality** | **Cost** | **Treatment outcomes** | | **Length of stay (LOS)** | **Infection and microbiology outcomes** |
| --- | --- | --- | --- | --- | --- | --- |
|  |  |  | **Duration of therapy** | **Dose** |  |  |
| Standiford et al., 2012 | 3% (2001) vs 2.5% (2010) | 45.8% decrease  (p=0.04) | NR | NR | 6.1 (2001) vs 5.5 (2010) days | NR |
| Cappelletty & Jacobs, 2013 | NR | 32.3% increase | 1–4 days longer without pharmacist | NR | NR | *Clostridium difficile* cases increased >3x. |
| Reed et al., 2014 | 19% vs 30% (p=0.11) | 25,697$ vs 31,457$ (p=0.25) | Decreased from 13.5 to 1.3 hours (p=0.04) | NR | 10 vs 11 days (p=0.68) | Diagnosis of candidemia increased 32% vs 48% (p=0.04) |
| Al-Somai et al., 2014 | NR | NR | No significant difference (p=0.476) | DDD/100 PD: NS | NR | NR |
| Whitney et al., 2018 | 38% vs 26% (p=0.100) | Initially decreased by 30%, then increased by 20% above the 5 years. | NR | DDD/100 PDs: decrease  in 2011 to 12, increase  in 2016 to 17 | Not changed significantly | NR |
| Pettit et al., 2019 | 30-day mortality:  %19 vs %26 (p=0.810) | NR | 4.8 vs 3.3 hours (p=0.580) | NR | 24 vs 18 days (p=0.280) | Average time to blood culture clearance: 3.7 vs 4.1 days (p=0.700) |
| Morris et al., 2019 | No significant difference (p=0.700) | 1,771.86$ vs 2,027.54$ (p=0.150) | NR | DDD/100 PDs: 30.5 vs 27.4 (p=0.050) | 6.10 vs 6.75 days  (p=0.250) | NR |
| Lachenmayr et al., 2019 | NR | NR | NR | DDD/100 PDs: 18 vs 16 | 23 vs 24 days, p=0.397 | NR |
| Samura et al., 2020 | 30-day mortality: 29.4% vs 60% (p=0.099) | 9,390.5$ vs 5,930.8$ (p=0.002) | DOT/1000 PDs: 6.0 vs 3.4 (p<0.001) | Optimal dose: 71.4% vs 100%, p=0.028 | NR | NR |
| Koh et al., 2020 | 5% for both groups (p=0.97) | 15,713$ vs 13,641$ (p=0.150) | Time to effective treatment: 13.9 vs 8.6 hours (p=0.290),  Time to optimum treatment 53.7 vs 38.4 hours (p<0.001) | NR | 11.0 vs 10.3 days (p=0.96) | Time to organism identification: 11 vs 4 hours |
| Ioannidis et al., 2020 | 28-day mortality: 42.3% | 35,306€ savings per year (p<0.05) | 18 days | DDD/100 PDs: 2.12 (2013) vs  2.29 (2016) | NR | NR |
| Kara et al., 2021 | 30-day mortality not significantly changed; %19, %15.6 and %27.5  (p=0.050) | NR | 13, 14 and 14 days (p=0.560)  Appropriateness: 64.0%, 57.7% and 75.2% (p=0.013) | NR | NR | Candidemia patients: 20.0%, 24.8%, and 19.1%) (p=0.193) |
| Markogiannakis et al., 2021 | No significant difference | 26.8% decrease (p<0.001) | No significant difference: 14.27 vs 16.49 days | DDD/100 PDs: 33.4 vs 25.5, p<0.001 | No significant difference: 5.19 vs 4.96 days | - |
| Moni et al., 2022 | 10% decrease (p=0.260) | NR | Treatment delay: 59.1 vs 8.3 hours (p=0.0001) | NR | ≥30days: 22% vs 33% (p=0.260) | Candidemia incidence rate: 1.38 vs 0.73 |
| Keck et al., 2023 | No significant difference (p=0.634) | NR | 4 vs 3 days (p=0.005) | NR | No significant difference (p=0.137) | Positive cultures for *Candida*: 9% vs 8% (p=0.832) |

DDD, Defined Daily Dose; DOT, Days of Therapy; NS, Not Specified; PD, Patient Days, NR = Not reported in the original study, NA = Not applicable

**Search Strategy**

**PubMed (MEDLINE)**

The search strategy was developed using combinations of Medical Subject Headings (MeSH) and free-text terms related to antifungal therapy, fungal infections, stewardship, and pharmacist involvement.

**Concept 1 – Antifungal therapy**

"antifungal agents"[Pharmacological Action] OR
"antifungal agents"[MeSH Terms] OR
antifungal*[All Fields]

**Concept 2 – Fungal infections**

"mycoses"[MeSH Terms] OR
mycoses[All Fields] OR
"fungal infection*"[All Fields]

**Concept 3 – Stewardship**

stewardship[All Fields]

**Concept 4 – Pharmacist**

"pharmacists"[MeSH Terms] OR
pharmacist*[All Fields] OR
pharmacy[All Fields]

**Final Search Strategy**

(("antifungal agents"[Pharmacological Action]
OR "antifungal agents"[MeSH Terms]
OR antifungal*[All Fields]
OR "mycoses"[MeSH Terms]
OR mycoses[All Fields]
OR "fungal infection*"[All Fields]))
AND
(stewardship[All Fields])
AND
("pharmacists"[MeSH Terms]
OR pharmacist*[All Fields]
OR pharmacy[All Fields])

**Limits**

- Language: English

**Scopus**

The following search strategy was used in Scopus:

TITLE-ABS-KEY
(
(antifungal OR "antifungal agents" OR mycoses OR "fungal infection")
AND
("antifungal stewardship" OR "antifungal stewardship program*"
OR "antifungal stewardship intervention*"
OR "antifungal stewardship team*"
OR "antifungal management program*"
OR "antifungal optimization")
AND
(pharmacist OR pharmacists OR pharmacy)
)

**Limits**

- Language: English

The reference lists of all included studies and relevant review articles were also manually screened to identify additional eligible studies that may not have been captured by the database searches.
